# Supplementary material for: Sustainable Development Goals and 2030 Agenda: Awareness, Knowledge and Attitudes in Nine Italian Universities, 2019
Source: Int J Environ Res Public Health. 2020 Dec 2;17(23):8968. doi: 10.3390/ijerph17238968 (PMC7730411; doi:10.3390/ijerph17238968)
Supplement: Supplementary file 1 [file ijerph-17-08968-s001.zip › Supplementary material - Questionnaire/EN_SDGs Questionnaire_03_06_2019.pdf]

Sustainable Development Goals and 2030 Agenda: a survey on awareness, knowledge and attitudes among Italian first-year University students for academic session 2018-2019.

Items marked with * are mandatory.

Introduction

Dear student,

we invite you to participate to the nonprofit multicenter study “Sustainable Development Goals and 2030 Agenda: a survey on awareness, knowledge and attitudes among Italian first-year University students for academic session 2018-2019” promoted by the University of Udine. The principal investigator (PI) of the study is Professor Silvio Brusaferro, with the cooperation of Dr. Laura Brunelli, Cecilia Smaniotto and Edoardo Ruscio from the Department of Medical Area of the same University.

This study has the aim to assess the level of awareness, knowledge and attitudes towards the Sustainable Development Goals (SDGs) and the 2030 Agenda, as well as the key themes for the realization of a sustainable future among the members of the academic community.

Participation is voluntary and will require 10 minutes ca. No compensation is provided for participants. You can take back your consent at any time by simply not ending the compilation, without providing any explanation and without losing any right or benefit; only data obtained from fully compiled questionnaires will be considered. In case of retracted consent, no new information will be collected and added to current data.

The questionnaire is available until **July 31st 2019**.

At the end of the compilation, you will be redirected to some supplementary contents about sustainability themes and initiatives for sustainable development activated on national level and by your University.

Since this is an observational study, no specific risks are identified for participants. By accepting to participate to the present study, you declare you have read and understood the information in this text; if you want to receive more explanations or information, you may contact: e-mail smaniotto.cecilia@spes.uniud.it; ruscio.edoardo@spes.uniud.it; laura.brunelli@uniud.it; telephone 0432 554767.

Thank you for your contribution,
the research team

By proceeding in the compilation, you accept the treatment of personal data as indicated by current regulations. You can take back your consent later as well, by simply not submitting the compiled questionnaire.

The questionnaire is conceived in order to avoid the collection of data that allow direct identification, even if potential, of participants. The EU Survey platform, developed by the European Community, does not collect further personal data from users, as indicated by its privacy policy available online at: https://ec.europa.eu/eusurvey/home/privacystatement. Cookies are deleted at the end of the session. The answers will be cancelled from the platform at the end of the online availability of the survey.

☐ * I accept yourTerms

Knowledge

Section 1: Knowledge

Please indicate, for the following items, your current level of knowledge of these topics. This is not an assessment questionnaire, but only a way to estimate the initial preparation on these themes!

Section 1A: Concepts

	Never heard about it	*Heard about it*	*Talked about it*	*Studied it at school*	*Autonomously informed about it*
* Sustainable Development Goals and 2030 Agenda	◯	◯	◯	◯	◯
* Planetary boundaries	◯	◯	◯	◯	◯
* Ecological footprint	◯	◯	◯	◯	◯
* Greenhouse effect	◯	◯	◯	◯	◯
* Resilience	◯	◯	◯	◯	◯
* Social gradient in health	◯	◯	◯	◯	◯
* Health inequalities	◯	◯	◯	◯	◯
* Determinants of health	◯	◯	◯	◯	◯

Section 1B: Indicators

	Never heard about it	*Heard about it*	*Talked about it*	*Studied it at school*	*Autonomously informed about it*
* Green GDP	◯	◯	◯	◯	◯
* Human Development Index (HDI)	◯	◯	◯	◯	◯
* Indicator of Sustainable Economic Welfare (ISEW)	◯	◯	◯	◯	◯
* Equitable and sustainable well-being (BES)	◯	◯	◯	◯	◯
* Genuine Progress Indicator (GPI)	◯	◯	◯	◯	◯
* Gross National Happiness (GNH)	◯	◯	◯	◯	◯

Section 1C: Documents and models

	Never heard about it	*Heard about it*	*Talked about it*	*Studied it at school*	*Autonomously informed about it*
* The Limits to Growth (1972 Report)	○	○	○	○	○
* Brundtland Report (1987)	○	○	○	○	○
* Montreal Protocol (1987)	○	○	○	○	○
* Kyoto Protocol (1997)	○	○	○	○	○
* Paris Agreement on climate change (2015)	○	○	○	○	○
* Doughnut Economy	○	○	○	○	○

Sources

Section 2: Sources

Please indicate, for the following items, from which sources you acquired the knowledge you currently have for the considered themes. If you don't know them, please select the option "I have no knowledge concerning this topic".

For each item you can select more than one option.

Section 2A: Concepts

	I have no knowledge concerning this topic	*School learning*	*Television*	*Newspapers/magazines/books*	*Web sources (e.g. websites, social networks, blogs)*	*Other sources*
* Sustainable Development Goals and 2030 Agenda	☐	☐	☐	☐	☐	☐
* Planetary boundaries	☐	☐	☐	☐	☐	☐
*Ecological footprint	☐	☐	☐	☐	☐	☐
* Greenhouse effect	☐	☐	☐	☐	☐	☐
* Resilience	☐	☐	☐	☐	☐	☐
* Social gradient in health	☐	☐	☐	☐	☐	☐
* Health inequalities	☐	☐	☐	☐	☐	☐
* Determinants of health	☐	☐	☐	☐	☐	☐

If you answered “Other sources”, could you indicate the sources from which you acquired your knowledge?

Section 2B: Indicators

	I have no knowledge concerning this topic	*School learning*	*Television*	*Newspapers/magazines/books*	*Web sources (e.g. websites, social networks, blogs)*	*Other sources*
***** Green GDP	☐	☐	☐	☐	☐	☐
***** Human Development Index (HDI)	☐	☐	☐	☐	☐	☐
***** Indicator of Sustainable economic Welfare (ISEW)	☐	☐	☐	☐	☐	☐
***** Equitable and sustainable well-being (BES)	☐	☐	☐	☐	☐	☐
***** Genuine Progress Indicator (GPI)	☐	☐	☐	☐	☐	☐
***** Gross National Happiness (GNH)	☐	☐	☐	☐	☐	☐

If you answered “Other sources”, could you indicate the sources from which you acquired your knowledge?

Section 2C: Documents and models

	I have no knowledge concerning this topic	*School learning*	*Television*	*Newspapers/magazines/books*	*Web sources (e.g. websites, social networks, blogs)*	*Other sources*
* The Limits to Growth (1972 Report)	☐	☐	☐	☐	☐	☐
* Brundtland Report (1987)	☐	☐	☐	☐	☐	☐
* Montreal Protocol (1987)	☐	☐	☐	☐	☐	☐
* Kyoto Protocol (1997)	☐	☐	☐	☐	☐	☐
* Paris Agreement on climate change (2015)	☐	☐	☐	☐	☐	☐
* Doughnut Economy	☐	☐	☐	☐	☐	☐

If you answered “Other sources”, could you indicate the sources from which you acquired your knowledge?

Attitudes

Section 3: Attitudes

For the following items, do you think university (and more specifically the degree course you are currently attending) should have a role in the acquisition of knowledge concerning the considered themes?

Section 3A: Concepts

	No, I do not consider it relevant for me	*No, I do not think university should teach me about it*	*It makes no difference for me*	*Yes, but only for personal wisdom*	*Yes, also for my professional future*
* Sustainable Development Goals and 2030 Agenda	○	○	○	○	○
* Planetary boundaries	○	○	○	○	○
* Ecological footprint	○	○	○	○	○
* Greenhouse effect	○	○	○	○	○
* Resilience	○	○	○	○	○
* Social gradient in health	○	○	○	○	○
* Health inequalities	○	○	○	○	○
* Determinants of health	○	○	○	○	○

Section 3B: Indicators

	No, I do not consider it relevant for me	*No, I do not think university should teach me about it*	*It makes no difference for me*	*Yes, but only for personal wisdom*	*Yes, also for my professional future*
***** Green GDP	○	○	○	○	○
***** Human Development Index (HDI)	○	○	○	○	○
***** Indicator of Sustainable Economic Welfare (ISEW)	○	○	○	○	○
***** Equitable and sustainable well-being (BES)	○	○	○	○	○
***** Genuine Progress Indicator (GPI)	○	○	○	○	○
***** Gross National happiness (GNH)	○	○	○	○	○

Section 3C: Documents and models

	No, I do not consider it relevant for me	*No, I do not think university should teach me about it*	*It makes no difference for me*	*Yes, but only for personal wisdom*	*Yes, also for my professional future*
* The Limits to Growth (1972 Report)	○	○	○	○	○
* Brundtland Report (1987)	○	○	○	○	○
*Montreal Protocol (1987)	○	○	○	○	○
* Kyoto Protocol (1997)	○	○	○	○	○
* Paris Agreement on climate change (2015)	○	○	○	○	○
*Doughnut Economy	○	○	○	○	○

Socio-demographic information

Section 4: Personal data

* Age

* Gender

- M
- F

* Region of provenance

- Valle d'Aosta
- Piemonte
- Liguria
- Lombardia
- Veneto
- Trentino-Alto-Adige
- Friuli Venezia Giulia
- Emilia-Romagna

- Toscana
- Marche
- Umbria
- Lazio
- Abruzzo
- Molise
- Puglia
- Basilicata
- Campania
- Calabria
- Sicilia
- Sardegna
- Other (including Republic of San Marino, Vatican City State)

* Attended University

- University of Milan (Vita-Salute San Raffaele)
- University of Brescia
- University of Catania
- University of Foggia
- University of Milan (Statale)
- University of Rome "La Sapienza"
- University of Turin
- University of Udine
- University of Verona

* Attended academic year

- I
- II
- III
- IV
- V
- VI
- Beyond the time prescribed for degree plan

* Previously attended secondary school

- Scientific high school
- Classical high school
- Linguistic high school
- Artistic high school
- Musical high school
- Social pedagogy high school
- Applied sciences high school
- Chartered accountant qualification
- Agricultural engineering qualification
- Primary school teaching qualification
- Hospitality training institute
- Technical institute
- School of art
- Other secondary school

* Have you attended any other degree course at University (also if not completed)?

- Yes
- No

If you have, did you complete the degree course?

- Yes
- No

* Didactic area of your degree course:

- Healthcare degrees
- Literature, history and art degrees
- Linguistic degrees
- Law and politics degrees
- Economic degrees
- Mathematics and computer science degrees
- Engineering and architecture degrees
- Other degree

* Have you ever attended any previous specific activity or course concerning sustainable development or the Sustainable Development Goals before?

- Yes
- No
